# The evo‐devo origins of the nasopharynx

**DOI:** 10.1002/ar.24950

**Published:** 2022-06-04

**Authors:** Roger Jankowski

**Affiliations:** ^1^ ORL Department Faculty of Medicine, University of Lorraine Nancy

**Keywords:** embryology, evo‐devo, evolution, nasopharynx

## Abstract

The process by which upper respiratory tract structures have changed over deep evolutionary time is, in part, reflected in the process of embryologic development. The nasopharynx in particular is a centrally located space bounded by components of the respiratory portion of the nasal cavity, cranial base, soft palate, and Eustachian tube. The development of these components can be understood both in terms of embryologic structures such as the branchial arches and paraxial mesoderm and through fossil evidence dating as far back as the earliest agnathan fish of the Cambrian Period. Understanding both the evolution and development of these structures has been an immeasurable benefit to the otolaryngologist seeking to model disease etiology of both common and rare conditions. This discussion is a primer for those who may be unfamiliar with the central importance of the nasopharynx both in terms of our evolutionary history and early embryological development of vital cranial and upper respiratory tract structures.

## 1 INTRODUCTION

The anatomy of the human  nasopharynx is well known but its embryological development is poorly understood. This is due to the complexity that presides over the development of the cephalic end of the embryo.

The existence of a link between the development of a species and the evolution of species stems from the observations of the first embryologists of the beginning of the 19th century, with the discovery of the germinal disc by Pander (1817), the gill slits by Rathke (1825) and the first works of comparative embryology carried out by von Baer which affirm that the egg is the universal structure at the origin of embryonic development (*De Ovi Mammalium and Hominis Genesi*, 1827). A century and a half later, in 1981, the Nobel Prize winner François Jacob wrote in Le jeu des possibles*: “*L'évolution ne tire pas ses nouveautés du néant. Elle travaille sur ce qui existe déjà […] opère à la manière non d'un ingénieur, mais d'un bricoleur* **.” But even today, no one knows how development is related to evolution.

* *The game of possibilities*.

**“Evolution does not take its novelties out of nothing. Evolution works on what already exists […] operates in the manner not of an engineer, but of a handyman”.

Since the beginning of the 21st century, evolutionary developmental biology or (evo‐devo) has attempted, by pursuing Darwinian logic, to integrate a century of genetic and molecular knowledge in the understanding of evolutionary changes (Gilbert, 2003; Hall, 2003, 2012). But if the links between the genotype and the phenotype of an individual remain poorly understood, it seems that by following the Lamarckian logic of increasing complexity, the chronological stages of the evolution of species make it possible to better understand the morphological stages of embryological development. There is thus a remarkable parallelism between the evolution of mammals, which are originally exclusively aquatic animals and only secondarily conquer the emerged lands first on four and then two legs, and the development of the nose and the middle third of the human face from which the anatomical complexity seems to derive (Jankowski, 2013). Thus evo‐devo science could alongside evolutionary developmental biology be strengthened by evolutionary developmental morphology, that is, the study of evolutionary morphological changes as a basis for understanding embryological, particularly human, development.

This article aims to provide an understanding of the human embryological development of the nasopharynx based on the chronological stages of the evolution of species.

## 2 THE NASOPHARYNX AS A CONSEQUENCE OF THE FORMATION OF A RESPIRATORY NOSE UNDER THE OLFACTORY NOSE

The nose of the first craniate vertebrates such as *Haikouichthys*, discovered in the Chengjiang deposit in China and dated around 530 million years ago (mya), is a priori an exclusively olfactory organ. It was formed of two sacs located in contact with the primary brain and which opened externally by an outer nostril. *Haikouichthys*' brain and olfactory, visual, and otic sense organs are housed in the forerunner of a skull, and traces of vertebrae are found on its fossilized remains (Shu et al., 2003).

The craniates were jawless fish (agnathans) that proliferated in the Ordovician and are known to us only through their fossils (with the exception of modern lampreys and hagfish). Fish with jaws (gnathostomes) suddenly appeared in the fossil record approximately 445 mya, alongside agnathans, without observation of transitional forms (Janvier, 2020). The only surviving models of these early vertebrates are the lamprey which is an agnathan fish and the lungfish which is a gnathostome fish. The oldest known fossils representing agnathan and gnathostome fish are dated to 360 mya (an early lamprey; Gess et al., 2006) and 390 mya (an early diabolepid or lungfish; Clairambaut & Janvier, 2021), respectively. The current chronology of their fossils therefore does not correspond with the supposed stages of functional evolution, both with regard to the evolution of the oral cavity and that of the olfactory organ.

**FIGURE 1:**
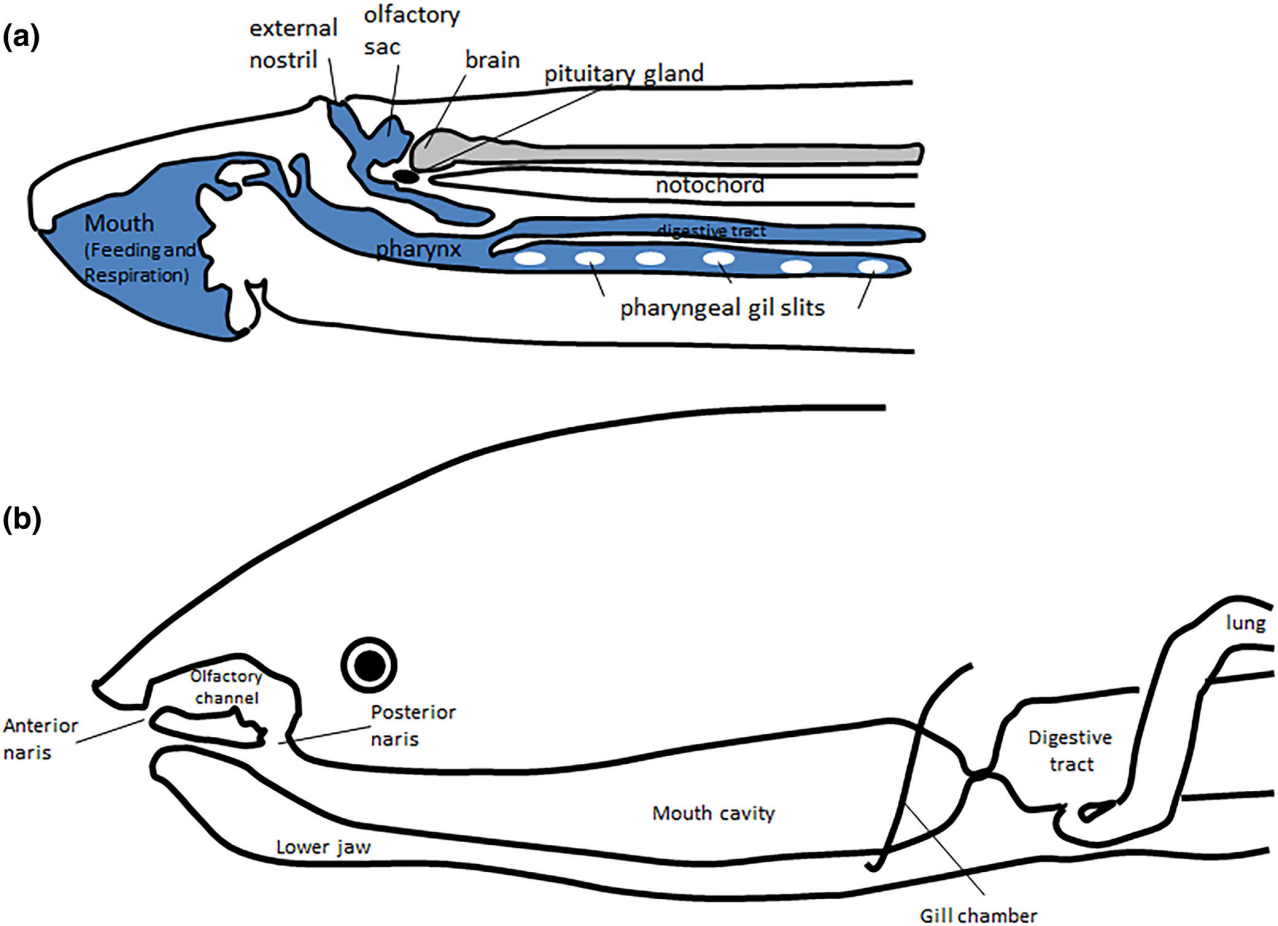
The olfactory nose of lampreys (agnathan fish) and Dipnoi (gnathostome fish). (a) Feeding, respiration and olfaction in the lamprey (adapted from Kuratani et al., 2001). (b) Lungfish olfactory channels (adapted from Derivot, 1984)

The olfactory organ of the lamprey (Kuratani et al., 2001) indeed seems to be intermediate between the condition of the first agnathan fish (i.e., *Haikouichthys*) and gnathostome fish such as the lungfish (Derivot, 1984). The olfactory organ (the nose) of the lamprey (Figure 1a) is formed of two sacs whose olfactory neurons are connected to the telencephalon. It opens to the outside by an external nostril, but its terminal appendix is blind and has no communication with the oro‐branchial respiratory organ. The olfactory organ of the lungfish (Figure 1b) opens through an external nostril but also into the oral cavity through an internal nostril (in the case of African and Australian lungfish, but not South American lungfish): the lungfish thus explores its olfactory environment by sucking water through the contraction of his oral floor muscles (oral pump). The lungfish's olfactory organ does not in any way participate in its breathing, whether branchial or pulmonary (Derivot, 1984).

**FIGURE 2:**
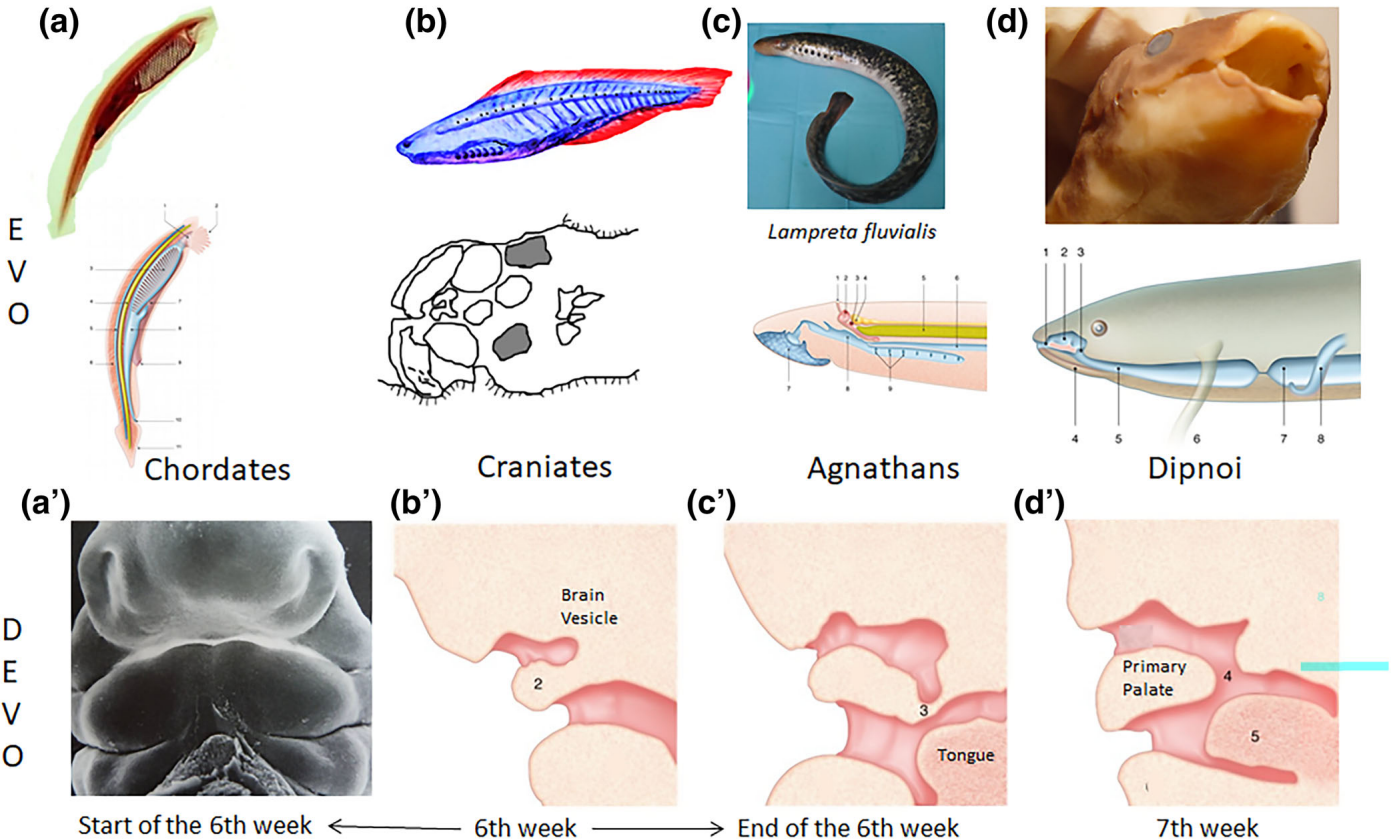
Parallel between evolution and development of the olfactory nose. (a) Chordate have scattered olfactory cells around the mouth which appear located in two placodes at the start of the sixth week in human embryo. (b) Human olfactory placodes invaginate in two olfactory pits by the sixth week forming olfactory pockets like in craniates. (c) An appendix develops toward the mouth at end of sixth week of human development like in the lampreys. (d) opening of the olfactory nose in the mouth at 7 weeks of human development as in some Dipnoi (gnathostome fish)

The formation of the nose initiated with the appearance of an olfactory organ among early fish. These stages of the functional evolution of the nose are largely re‐capitulated during human embryological development (Figure 2). The process begins with the olfactory placodes invaginating into olfactory wells whose neurons connect to the olfactory bulbs, and the olfactory wells open secondarily into the oral cavity. This occurs by rupture of the oro‐nasal membrane of Hochstetter, behind the primary palate in which will develop the premaxillary bones, bearing the four incisor teeth (Larsen, 2001).

**FIGURE 3:**
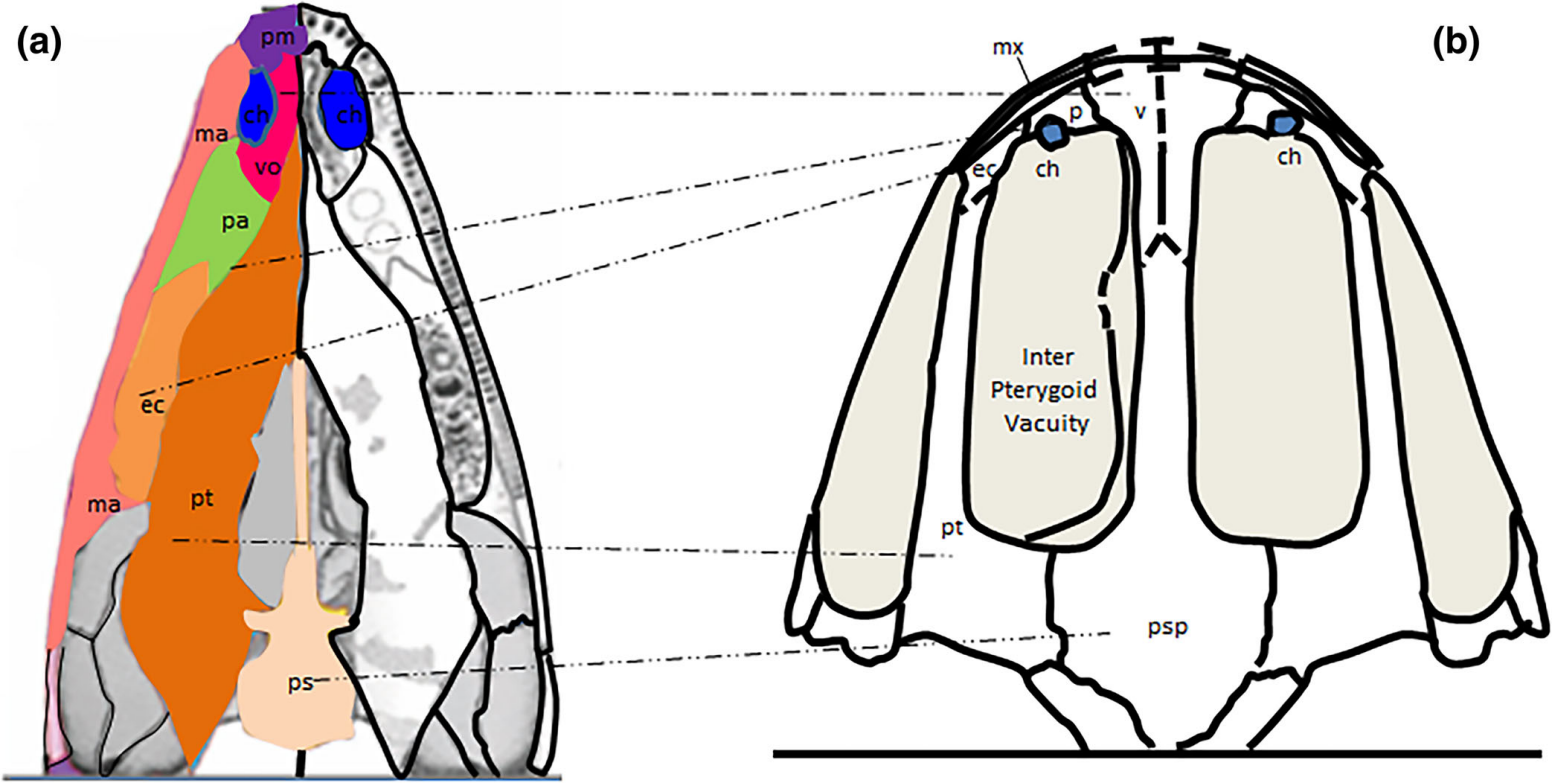
Preservation of the palatal bones from the paleozoic early tetrapods to the modern amphibians. (a) Anatomy of the secondary flat palate in paleozoic early tetrapods (adapted from Kimmel et al., 2009). (b) Preservation of the early tetrapod palatal bones in frog's palate, where they surround two large inter‐pterygoid vacuities for the orbits

The primitive Paleozoic tetrapods, from the lungfish *Eusthenopteron* (385 mya) through the amphibian *Ichtyostega* (365 mya) to the terrestrial tetrapod *Pantylus* (255 mya), have developed in the mouth, behind the primary palate, a secondary bony palate which is attached to the base of the skull. It is flat and formed of five bones per side (only the shape and size of which vary according to the species), arranged in two rows, one internal (vomer anterior, pterygoid posteriorly) and the other external (anterior to posterior palatine, ectopterygoid, and palatal process of the maxilla; Figure 3a; Kimmel et al., 2009). The great Permian–Triassic extinction that occurred around 252 mya has left us their fossils only. Modern amphibians did not appear in fossil records until after a fossil gap of about 30 my. The anatomy of current amphibians shows us, however, the complete conservation of the bones of the secondary palate with, as in primitive tetrapods, adaptations in shape and size depending on the species (Figure 3b). Functionally, the nose of current amphibians remains essentially an olfactory nose both in water and in the air but can function in pulmonary respiration. It is through the oral pump that air is inhaled or drawn through the nostrils and then “pushed” from the mouth into the lungs, forcing the glottis (Barker, 2000).

**FIGURE 4:**
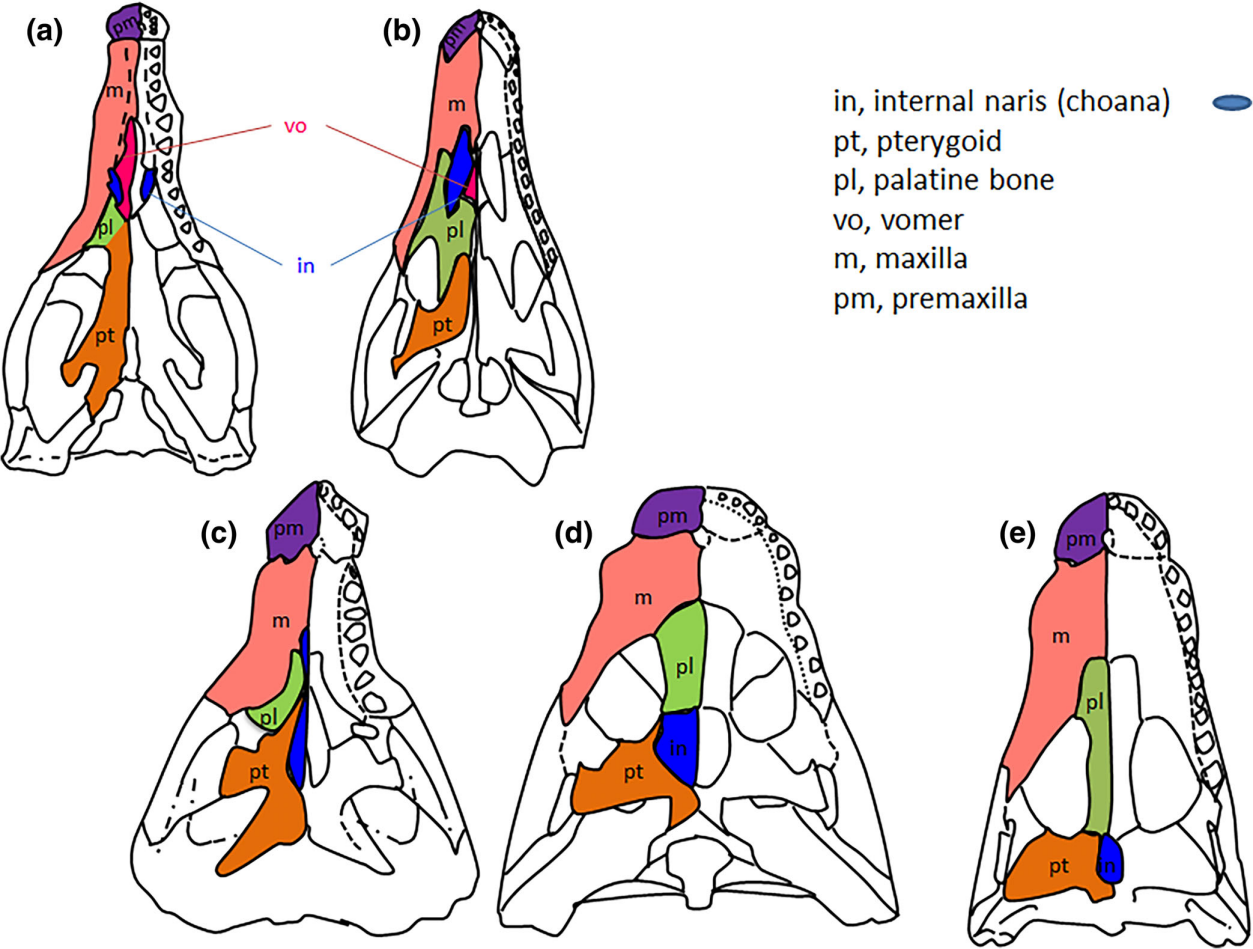
Evolution of the secondary palate in crocodilians. Posterior shift of the internal choanae and simultaneous opening of the secondary nasal passage. Adapted from Russel and Wu (1997)

**FIGURE 5:**
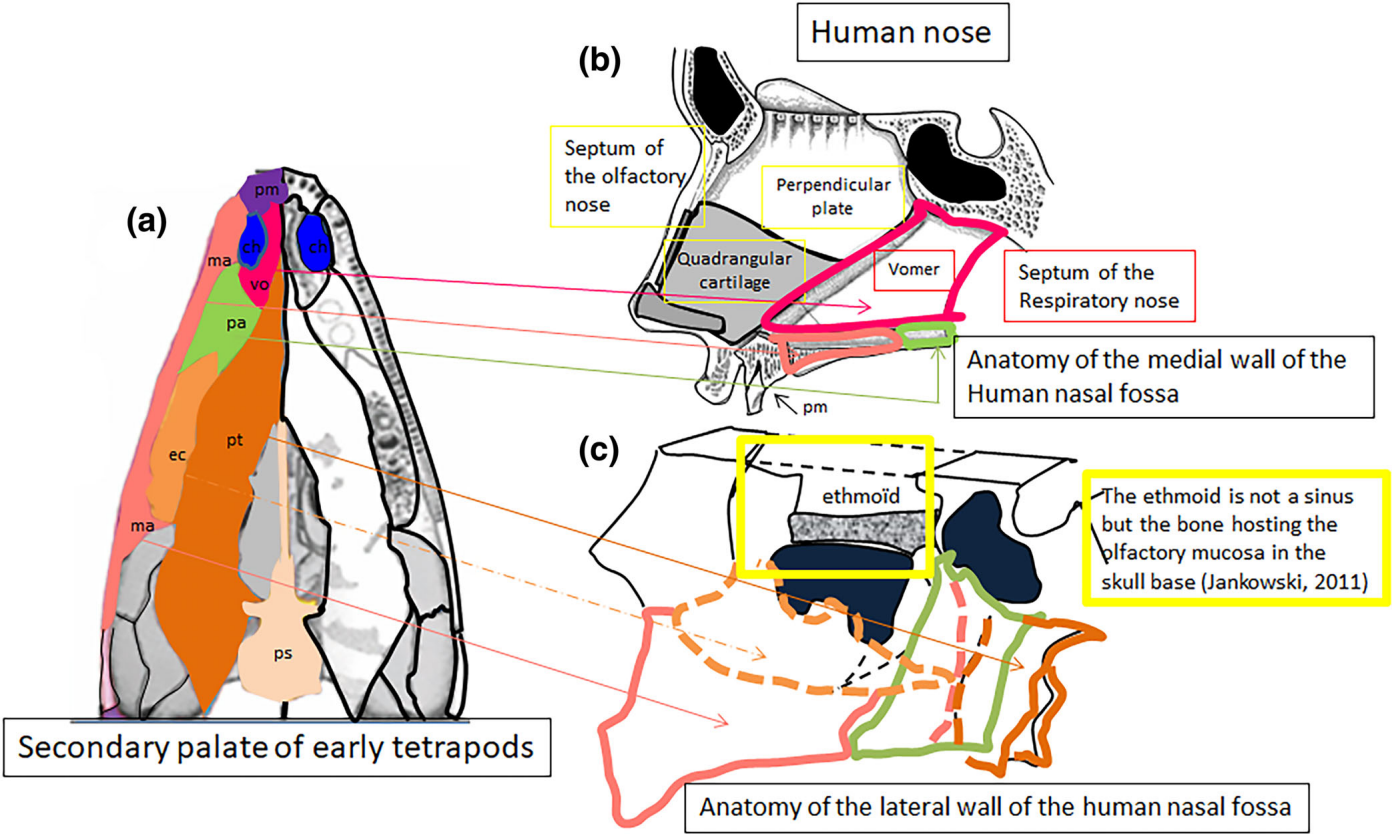
Origin of the human respiratory nose. The secondary respiratory nose is walled in by the primary palatal bones of the early tetrapods

It is probable that, in the tetrapods who adapted to an essentially terrestrial existence, they still returned to the water to lay eggs but use of the nasopulmonary air respiration became predominant. The location of the internal nostrils in the anterior part of the oral palate however, disrupted the functions of the primary mouth, which provided the respiratory, food and prey capture functions all at the same time. The slow and gradual displacement of the internal nostrils backward, from their primitive position behind the primary palate to that behind the secondary palate, can in fact be followed during the evolution of the palate of crocodilians between 252 and 65 mya (Figure 4; Russel & Wu, 1997/98). At the same time, the bones of the secondary palate of crocodilians were reshaped to form the walls of two respiratory corridors, independent of the oral cavity, under the olfactory nose. The walls of the respiratory nose of mammals (Moore, 1981), as well as those of the human respiratory nose (Figure 5), are currently formed by the adaptive arrangement of the bones of the secondary palate of primitive tetrapods in two corridors below the olfactory nose. Among most mammals, the olfactory nose remains separated from the respiratory nose by the transverse lamina while anthropoid primates exhibit a lack of this transverse plate as they are microsmatic by contrast (Smith & Rossie, 2006). This wrongly suggests that the human nose is a single organ. The olfactory nose, located above the respiratory nose, is in fact formed by the basicranial receptacle of the olfactory mucosa that is the ethmoid bone (Jankowski, 2011; Jankowski, Perrot, et al., 2016; Jankowski, Rumeau, et al., 2016) and by two ducts posteriorly positioned side by side, differentiated from the walls of the embryonic olfactory wells in several anatomical elements (alar cartilages, septolateral cartilages [Varoquier et al., 2021], and fibrous fascia) altogether constituting the olfactory fascia (Jankowski, Perrot, et al., 2016; Jankowski, Rumeau, et al., 2016).

The anatomy of the human vomer (Figure 5b) is probably the most suggestive of the formation of the respiratory nose under the olfactory nose (Botti et al., 2017). The two vomers which in the primitive tetrapods bordered horizontally in a paramedian position the two internal nostrils behind the premaxillae (Figure 5a) are found fused into a single vertical bone in the human nasal septum, under the two constituent elements of the olfactory septum formed by the perpendicular plate of the ethmoid and the quadrangular cartilage (Figure 5b). The vomer appears to have separated the body of the sphenoid from the surface of the secondary palate, which now appears only formed by the hyper‐developed palatal processes of the maxillae and the horizontal processes of the palatine (Moore, 1981), which itself could be functionally secondary to the development of grinding or chewing more and more solid food thanks to the maxillary teeth (Thomason & Russell, 1986). The planar development of the maxillary palatal processes would have pushed not only the vomers to verticalize under the septum of the olfactory nose, but also the powerful pterygoid bones to verticalize under the body of the sphenoid as well as the palatine bones to bend at right angles, and the ectopterygoids to curl under the transverse processes to form the inferior turbinates (Figure 5c).

Thus, the respiratory nose would have developed under the ethmoidal and sphenoidal base of the skull, transposing the primitive internal nostrils, which only persist in the vestigial state in the incisive canal behind the premaxillae, near the glottis of the pulmonary tract. The mouth, thus short‐circuited and freed from its respiratory function, could then ensure its food and capture functions in quadrupeds. But, the evo‐devo scenario of the formation of the respiratory nose, anterior skull base, and midface (Jankowski, 2013) does not provide a full explanation for the formation of the nasopharynx.

## 3 FORMATION OF THE NOSE AND PHARYNX

The origin of the pharynx appears, on the evo‐devo plan, entirely derived from the branchial apparatus, on condition that the mouth is not formed from the first branchial arch or from the (probably wrongly named) “maxillary and mandibular arches.” Under this condition, the first three branchial arches participate through their respective cartilage in the formation of the three ossicles of the middle ear, and the tubal orifices that open onto the lateral walls of the nasopharynx. Under these conditions, the nasopharynx appears as a mixed organ derived both from the respiratory nose and from the cephalic end of the embryonic pharynx.

### 3.1 The mouth originates from the embryonic stomodeum

**FIGURE 6:**
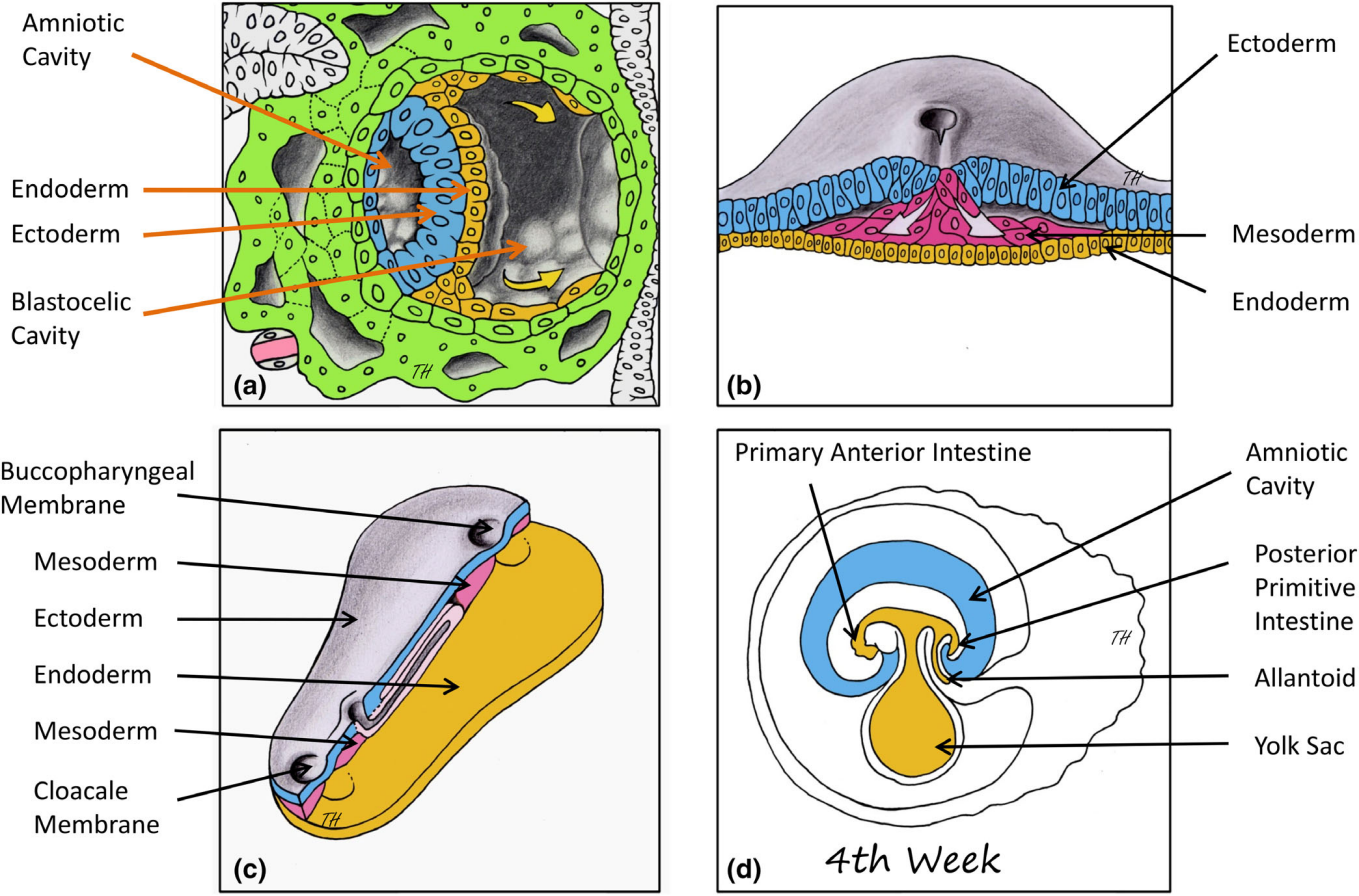
Human embryological development of the primitive intestine (adapted from Larsen, 2001). (a) the endoderm of the didermic embryo lines the blastocelic cavity which becomes the yolk sac. (b) Gastrulation transforms the didermic embryo into a tridermal embryo. (c) Formation of the buccopharyngeal and cloacal membranes. (d) Endodermal formation of the anterior and posterior ends of the primary intestine

During gastrulation and the formation of the tridermal embryo (Figure 6b), the formation of the mesoblastic layer does not interpose between the epiblastic and endoblastic layers in two zones which become the buccopharyngeal and cloacal membranes (Figure 6c). The endoderm lined the yolk cavity of the didermic embryo (Figure 6a). This yolk cavity then gives rise in the tridermal somatic embryo to the anterior and posterior ends of the primitive intestine, the blind ends of which remain formed by the endoderm portions of the buccopharyngeal and cloacal membranes (Figure 6d).

**FIGURE 7:**
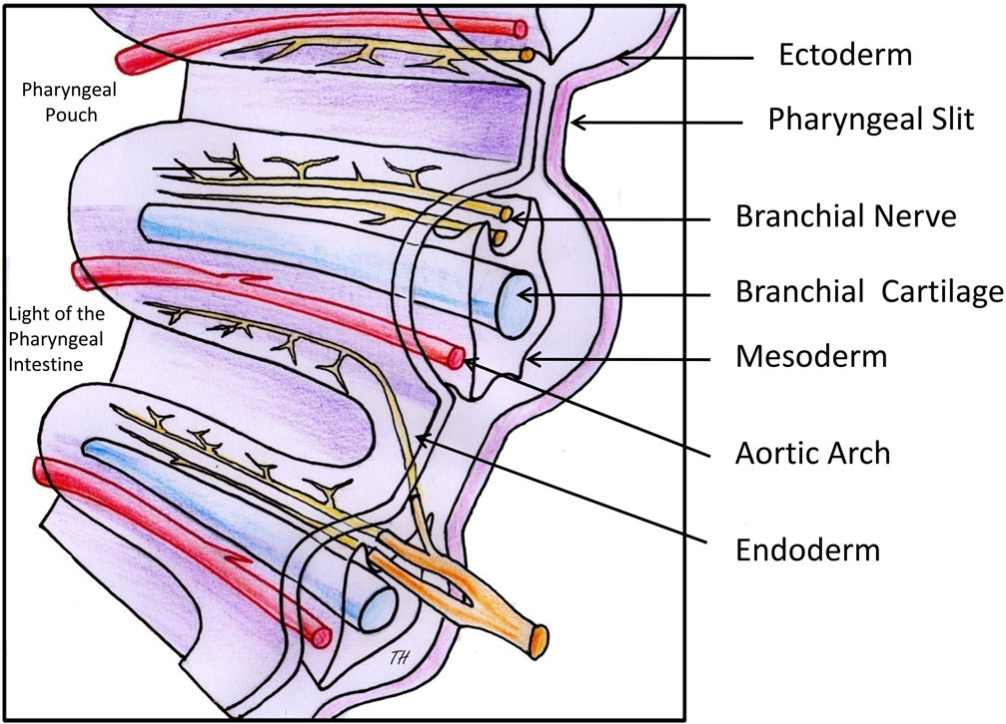
Diagram of the organization of the pharyngeal arches

On day 22 of human development, five pairs of pharyngeal arches (equivalent to the gill arches of primitive vertebrates) appear on either side of the pharyngeal intestine. Each arch is formed by an external coating of ectoderm, an internal coating of endoderm and a mesodermal axis in which differentiate a cartilage and myoblasts, is supplied by an aortic arterial arch and innervated by a cranial nerve (V, VII, IX, and X, respectively). The arches are separated by constrictions forming on the outside the pharyngeal slits lined with ectoderm and internally the pharyngeal pouches lined with endoderm (Figure 7). It is classically assumed (Larsen, 2001) that the cartilage of the first arch, split into two, is the origin of the upper and lower jaws in primitive vertebrates, but that in humans the jaws are almost entirely of membranous origin and that the cartilage of the first arch is at the origin of the malleus and incus of the middle ear and that of the second arch at the origin of the stapes, the stylohyoid ligament and the superior border of the hyoid bone.

It is classically accepted that the human face is formed from the five processes surrounding at the fourth week the stomodeum, a depression closed in depth by the bucco‐pharyngeal membrane and which thus appears as the precursor of the mouth. It has been argued (Larsen, 2001) that the so‐called “maxillary and mandibular” processes are at the origin of the upper and lower jaws. The frontal process housing the encephalic outline forms the roof of the stomodeum. The frontal process ectoderm thickens at several specific sites called pituitary, olfactory, optic, and otic placodes (Larsen, 2001). This classic conception of the development of the human face can, however, be called into question by an evo‐devo approach, which suggests that the so‐called “maxillary and mandibular” processes do not participate in the mouth but in the rhinopharynx formation.

### 3.2 Jawless fish (agnathans) precede fish with jaws (gnathostomes)

**FIGURE 8:**
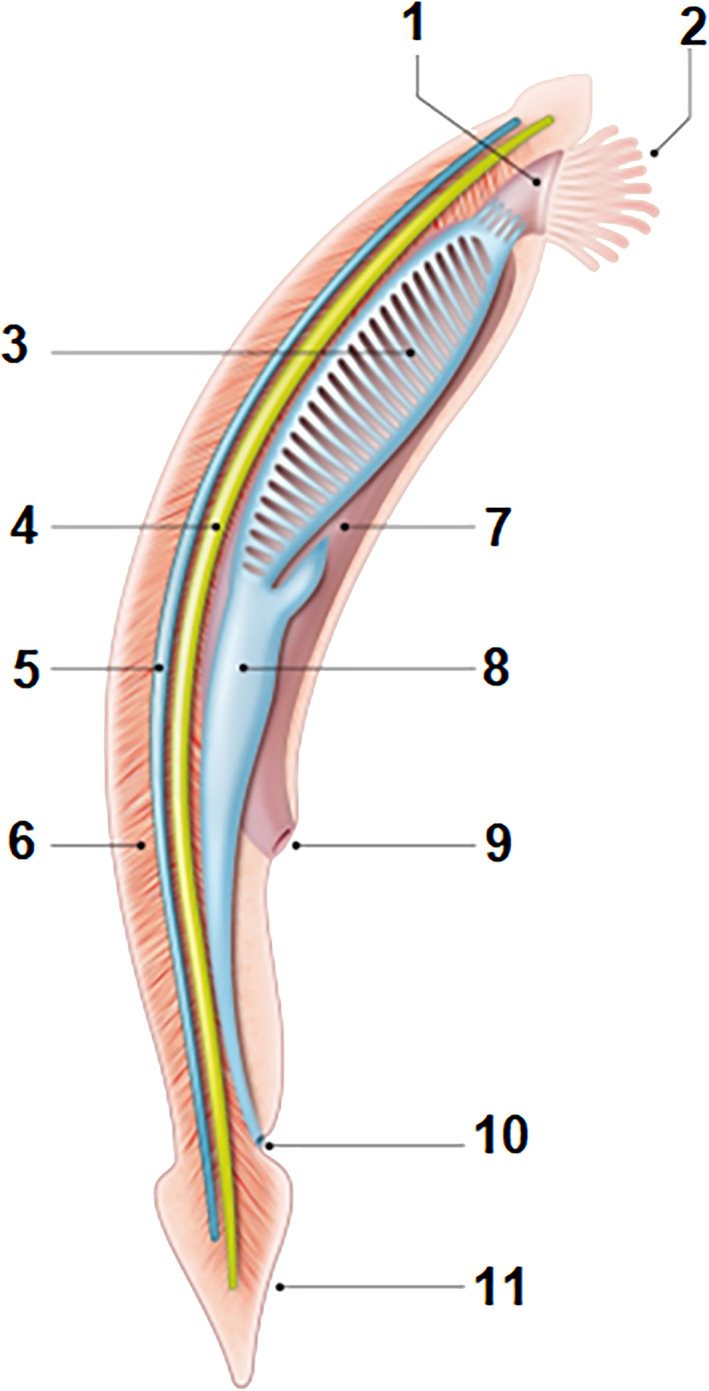
Anatomy of a cephalochordate (amphioxus). Cephalochordates are characterized by a cartilaginous notochord (4) which supports a nerve chord (5). They live silted up at the bottom of a body of water bent against the current. Water enters a pharyngeal mouth (1) surrounded by tentacles (2) and passes through the gills (3) where oxygen is taken. Suspended food particles are trapped by cilia which drain them into the intestine (8). The gill slits do not open outward but into a peribranchial cavity or atrium (7) which opens outward through an abdominal pore or atriopore (10). A short tail (11) allowing movement by contraction of the segmental muscles (6) extends the body beyond the anus (10) 1, mouth; 2, tentacles, 3, pharyngeal slits; 4, dorsal notochord; 5, dorsal nerve cord; 6, segmental muscles; 7, atrium; 8, intestine; 9, atriopore; 10, anus; 11, tail

The mouths of the oldest supposed ancestors of vertebrates, which lived in the Cambrian age such as *Haikouichthys* approximately 530 mya, have no jaw and resemble the branchial pharynx of chordates whose cilia mobilize respiratory water and filter food particles suspended in water (Shu et al., 2003; Figure 8).

Ostracoderms, the fossils of which are found around 490 mya in the Ordovician strata following the Cambrian period, are extinct agnathan fish, characterized by a bony exoskeleton encompassing the head and chest girdle. Moore's hypothesis is that some of these primordial dermal bones would have been preserved in the formation of the skull and face of vertebrates, among which he ranks the bones of the oral palate (Moore, 1981).

The first jawed fish appear in the fossil record around 445 mya, during the transition between Ordovician and Silurian periods. We do not know of anatomical forms of transition between the agnathan ostracoderms and the first gnathostome fishes. In fact, we typically find in strata containing fossil vertebrates from the Silurian period, alongside an abundance of agnathan fish, a few specimens of gnathostome fish, mainly acanthodians and osteichthyans (Janvier, 1996, fig. 1.3, p. 4), with none representing any anatomical forms of transition. In fact, gnathostomes remain extremely rare until the end of the Silurian or even the beginning of the Devonian when they underwent adaptive radiation approximately 420 mya. It is still not known why a mouth with articulated jaws suddenly appeared in the fossil record around 410 mya. However, this was probably one of the fundamental factors of vertebrate radiation, since over 99% of present day vertebrates have jaws and all agnathans except lampreys and hagfish have become extinct (Brazeau & Friedman, 2015).

There has long been a consensus on the hypothesis that jaws were derived from the first branchial arch or maxillo‐mandibular arch. But it has conversely been proposed that the maxillo‐mandibular arch could never have been a branchial arch, and that the oropharyngeal region would simply have adapted to the morphology of the posterior branchial arches with respiratory function when the first gnathosomes would have acquired a partition between dorsal portion and ventral portion (Janvier, 1996, 2020).

**FIGURE 9:**
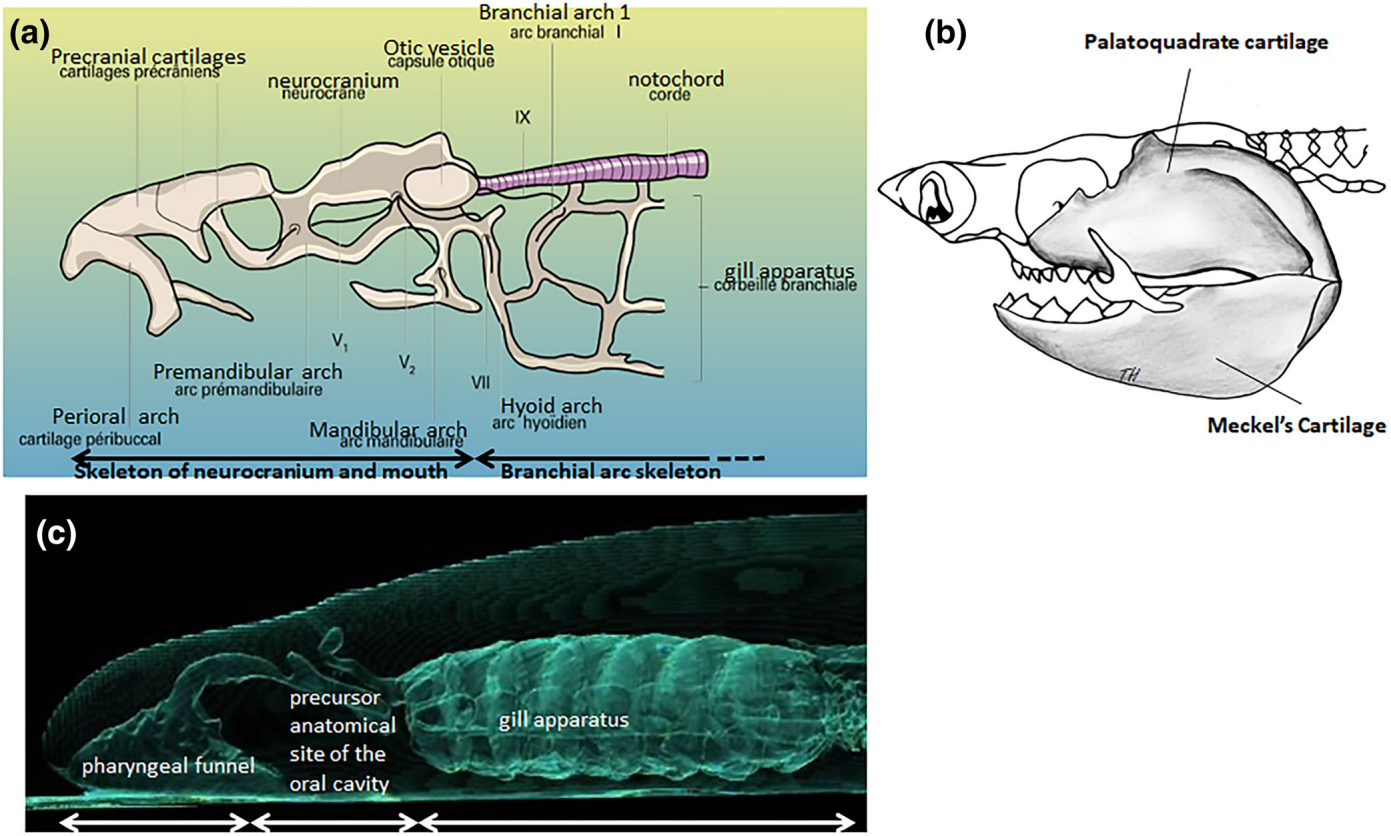
Evolutionary origin of the jaws. (a) Diagram of the cephalic skeleton of the adult lamprey (after Marinelli & Strenger, 1954). (b) Shark jaws represent the study model for all gnathostomes. (c) CT scan of the cephalic end of a lamprey from the Gironde estuary (France) (Guilloz Radiology Department, CHRU Nancy, France)

The oropharyngeal skeleton of the adult lamprey reveals the existence of a cartilaginous structure which could be a satisfactory model, in terms of functional evolution, for the origin of jaws. Indeed, this structure may represent a primitive homologue of the mandible as the skeleton of the cartilaginous neurocranium and oral cavity is clearly distinct in lamprey from that of the branchial arches (Figure 9a; Marinelli & Strenger, 1954).

Although the structure of the jaws shows great diversity among vertebrates, their organization is fundamentally always the same: a moveable lower jaw opposing an upper jaw more or less attached to the cranium. In the shark (cartilaginous fish), whose jaws represent the study model for all gnathostomes, the cartilage of the lower jaw or Meckel's cartilage articulates with the upper jaw cartilage or palatoquadrate (Figure 9b). Comparison of the lamprey and shark skeletons actually suggests me that the cartilage called the premandibular arch in the agnathic lamprey might be the equivalent of the palatoquadrate cartilage in the gnathostome shark, and that the cartilage called the mandibular arch in the lamprey might be the equivalent of Meckel's cartilage in sharks. It seems also probable to me that the structure identified as the opthalmic nerve, which crosses the premandibular arch in the lamprey on Figure 9a, is actually the maxillary nerve (CN V_2_), and that the structure identified as the maxillary nerve which crosses the mandibular arch is in reality the mandibular nerve (the V_1_ in connection with the optic capsule would not be visible on this diagram). A CT scan of the cephalic end of the lamprey makes it possible to visualize the position of its skeleton and the precursor anatomical site of the oral cavity between the pharyngeal funnel and the gill apparatus (Figure 9c).

It is of note for paleontologists that the palatoquadrate is attached primitively to the cranium at three points: behind the nasal capsules, at the base of the orbital cavity and behind the orbital cavity. These three points of attachment exist only in primitive elasmobranchs and acanthodians (the group of extinct osteocartilaginous fishes). In many gnathostomes, the palatoquadrate tends to be increasingly mobile, only articulating with the neurocranium through the two most anterior joints. Conversely, the palatoquadrate can lose all its mobility by fusing with the neurocranium, as in lungfish or tetrapods (Janvier, 2020).

The oral cavity of gnathostomes is schematically a chamber in the form of a rectangular parallelepiped, the frame of which is formed laterally by two pairs of symmetrical bones hanging under the base of the skull, which derive from Meckel's cartilages for the mandibular bones and palatoquadrate cartilages for the jawbones. The two palatoquadrate cartilages are usually fused medially to the base of the anterior cranium while the Meckel cartilages are articulated below the base of the middle and lateral cranium. The two jaws articulate with each other, uniting the upper and lower tooth rows.

**FIGURE 10:**
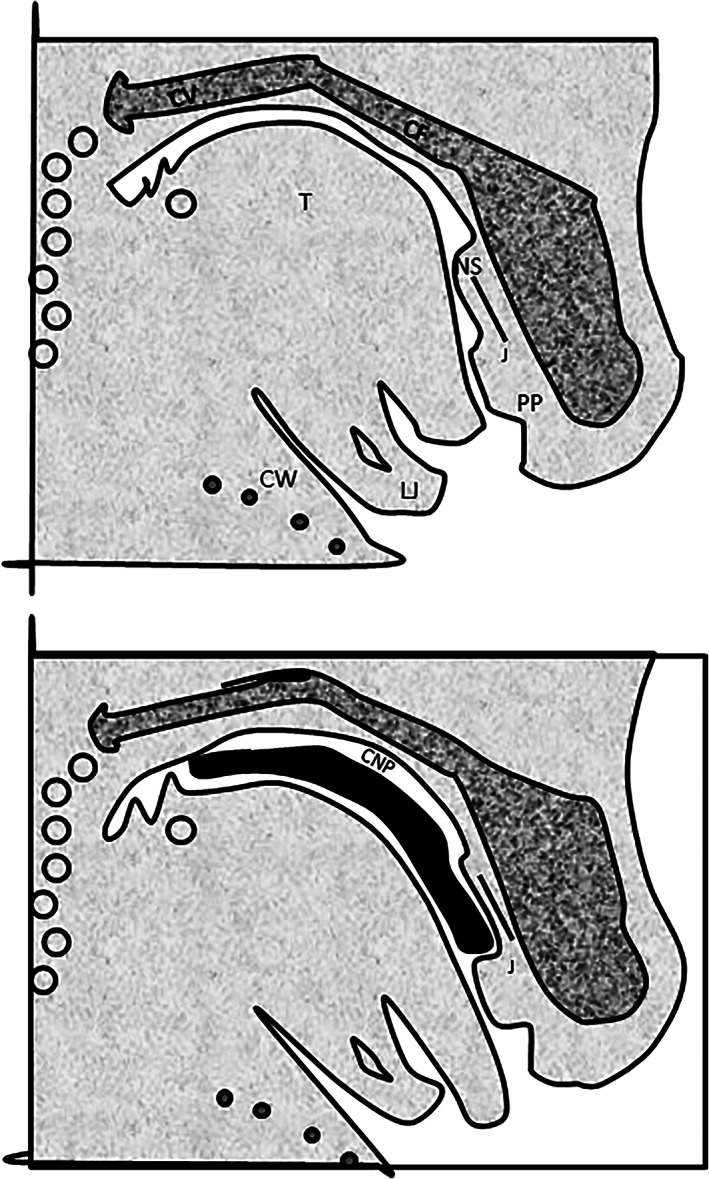
Hypothetic palato‐square cranial base. (a) Diagram drawn from tracings of sagittal sections of 16.3 day rat fetuses. Note the relationship between the arched tongue (T) and cranial floor posteriorly (CF) (i.e., the palatoquadrate); and the bulge of the primary palate (PP) and nasal septum (NS) anteriorly, which appear to be directing the tongue tip out of the oral cavity. The angulation of the cranial base and cervical vertebrae (CV) and the relationship between the lower jaw (LJ) and chest wall (CW) should also be noted. J, Jacobson's organs. (b) Diagram drawn from tracings of sagittal sections of 16.5 day rat fetuses. Note the elevated palatal shelves (solid black) and the common nasal passage (CNP). Space for the common nasal passage (compare figure a) is provided by protrusion of the tip of the flattened tongue out of the oral cavity, such protrusion obviously being facilitated by the sloping bulge of the primary palate and nasal septum. The role of the nasal septum as a “stop” for the secondary palate is obvious, and it should also be noted that there has been no change in the angulation of the cranial base or cervical vertebrae, and no change in the relationship of the lower jaw and chest wall. (Adapted from Ferguson, 1978)

During embryogenesis in mammals, the first movements detectable in the orofacial region seem to involve the active opening of the mouth by depressing the mandible, which is then recessed under the upper jawbones (retrognathia; Herring, 1985). These lowering movements of the jaw could be attributed to the maturity of the digastric, mylohyoid, and perhaps also infra‐hyoid muscles (Humphrey, 1971). At this stage, the only maxillary structures visible are the palatoquadrate cartilages lying at the underside of the brain and thus forming a primordial floor to the endocranial cavity. These movements appear as the barely formed tongue leans back against the base of the skull (i.e., probably the palatoquadrates in an evo‐devo understanding), behind the primary palate (which is the floor of the olfactory nose; Figure 10; Ferguson, 1978). Along with this muscular activity, the mandible grows forward with correction of the initial retrognathia, the contours of the posterior nasal cavity boundaries appear in the jawbone, and the tongue is lowered. It can be assumed that the contractile maturity of the muscles of the oral floor (digastric, mylohyoid, and perhaps also infra‐hyoid) induces the formation of a network of synapses in the central nervous system which in turn coordinate the arrangement of all the elements of the perioral region. By lengthening forward, the mandibles probably pull the palatoquadrate cartilages that, by moving away from each other, open the way to the olfactory pits and to the development of the ethmoid bone, which then enters into its final position at the anterior base of the skull. At the same time, the palatoquadrate cartilages are reshaped and form on each side of the tongue their palatal processes (which by straightening will form the secondary palate and the respiratory nose), and on either side of the olfactory pits their frontal or ascending processes (which delimit the anterior nasal piriform aperture).

### 3.3 The first three pharyngeal arches participate in the formation of the middle ear and nasopharynx

The evo‐devo hypothesis is that the three ossicles of the human middle ear probably derive from the cartilage of each of the first three pharyngeal arches. The muscles of the middle ear and of the nasopharynx, in particular the muscles of the soft palate, would thus probably also find their origin in the functional differentiation of the mesoderm of the first three (or four) branchial arches. The formation of the respiratory nose, as described previously, does not explain the origins of the human soft palate, a structure functionally essential to the demands of breathing, deglutition, hearing and articulated speech. The gular valve of crocodilians, often used as a model of the development of the respiratory nose, is a membranous flap devoid of muscle which allows them in immersion to isolate completely the mouth from the respiratory tract. The crocodilian gular valve, however, is not homologous with the mammalian soft palate and is probably not a precursor of the muscular palate of mammals (Putterill & Soley, 2006).

#### 3.3.1 The formation of the middle ear is secondary to that of the inner ear among vertebrates

**FIGURE 11:**
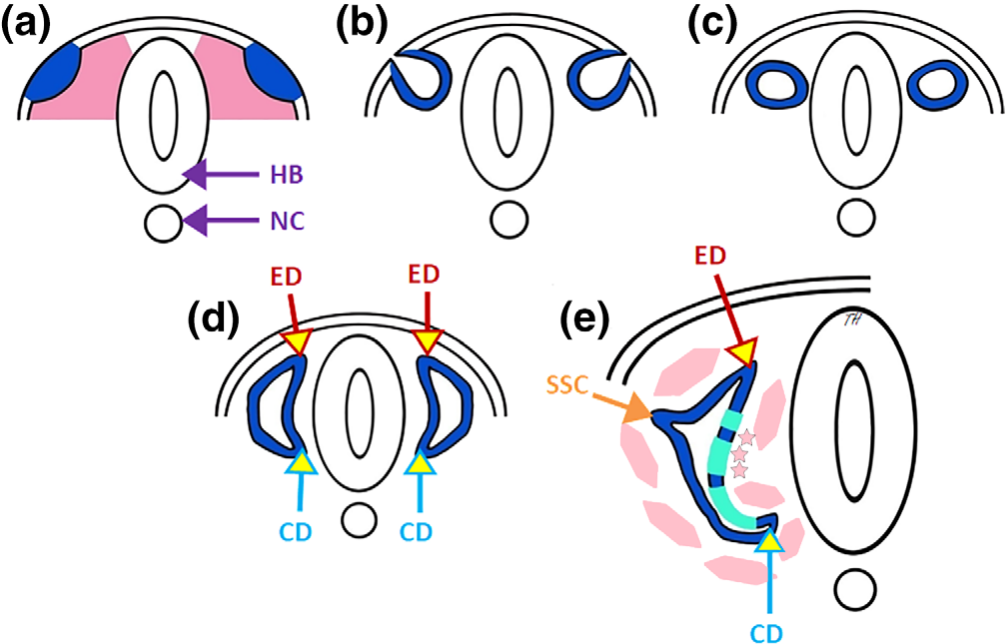
Formation of the inner ear of vertebrates (adapted from Barald & Kelley, 2004). (a) Appearance of otic placodes in the form of ectodermal thickening (blue) opposite the rhombocephalic brain (HB) (NC, notochord). (b) Invagination in cupshape of the otic placodes toward the rhombocephalus. (c) Individualization of the otic vesicle of the ectoderm and formation of the statoacoustic ganglion at its ventral pole. (d) Formation of the endolymphatic duct (ED) at the dorsal pole and of the cochlear duct (CD) at the ventral pole of the otic vesicle. (e) Differentiation of baro‐sensory receptors at the ventral pole (green), and appearance of semicircular canals (SSC) at the lateral pole of the otic vesicle; cochlear duct (CD) coiling and mesoderm differentiation (pink)

As with the nose, the first visible sign of ear formation in the human embryo is the appearance of an ectodermal placode at a specific site, at the level of rhombomeres 5 and 6 of the hindbrain (rhombencephalon; Figure 11a). This otic placode, made up of barosensory cells, invaginates toward the central nervous system to form a cup (Figure 11b) then a neuroepithelial sac buried under the ectodermal surface called the otic vesicle (Figure 11c). A few cells immediately detach from the ventral pole of the vesicle to form the statoacoustic ganglion (Figure 11c); their dendrites remain connected to the cells of the otic vesicle while their axons enter the central nervous system and probably induce the differentiation of the auditory centers there. The otic vesicle then emits a dorsal prolongation forming the endolymphatic duct (Figure 11d) which ends in the endolymphatic sac before undergoing a ventral prolongation to form the cochlear duct (Figure 11d). The level of the endolymphatic sac is also where exchanges with the cerebrospinal fluid form endolabyrinthine fluids. The otic vesicle then turns into an elaborate labyrinth housing the differentiated receptors of the vestibular and auditory systems. In its adult form, six areas of mechano‐electric transduction epithelium linked together by a simple cuboidal epithelium have become individualized in the membranous baro‐sensory labyrinth. The saccule and utricle sense linear and angular accelerations, the three vestibular ridges of the semicircular canals sense position in space, and the organ of Corti housed in the cochlea perceives sound vibrations. The mesoderm around the otic capsule organizes parallel to this differentiation of barosensory receptors (Figure 11e; Barald & Kelley, 2004).

#### 3.3.2 The middle ear adapted for hearing in terrestrial environments

Without the middle ear, 99% of sound energy is reflected by the body surface (van Bergeijk, 1966). Modern amphibians do not have an outer ear but have a middle ear with a large eardrum that is clearly visible behind the eye.

The first Devonian amphibians such as *Eusthenopteron*, dated to approximately 385 mya, could possess the earliest, most ancestral middle ear morphology among terrestrial vertebrates. The *Eusthenopteron* fossil presents a diverticulum of the first pharyngeal pouch (or spiracular pouch; Jarvik, 1954), the blind bottom of which comes into contact with the otic vesicle (van Bergeijk, 1966). Modern fish such as Anabantidae have similar aerial diverticula which have been shown to play a role in hearing of these fish (Schneider, 1942). It is therefore speculated that the *Eusthenopteron* spiracular diverticulum may contain air. This diverticulum appears wedged between the otic vesicle internally and two dermal bones externally, the parietal and the squamosal, which may have been linked together in life by ectodermal ligament at the level of the blind fundus of the spiracular diverticulum. Thus were affixed one on top of the other a sheet of endodermal origin on a sheet of ectodermal origin forming the external wall of the blind bottom of the spiracular diverticulum. Its condition may thus be described as a primordial tympanic cavity closed on the outside by a primordial tympanic structure (van Bergeijk, 1966).

It was argued by van Bergeijk (1966) that the hearing of *Eusthenopteron* could have been equivalent in water and in air among Paleozoic tetrapods, assuming the existence of a possible columellar effect between the primordial tympanic membrane and the otic capsule of *Eusthenopteron*. The only plausible hypothesis as to the origin of this columella then led him to propose a derivative of the hyomandibular bone (bone of the second pharyngeal arch). It was, however, neither useful nor necessary for *Eusthenopteron* to have the same hearing in water and air, and an absence of a stapes in the primordial middle ear model can be expected when considering the case of *Sechellophryne gardineri*.

*S. gardineri* is a tiny frog isolated for 47–65 mya in the Seychelles archipelago after the fracture of the supercontinent Gondwana 160 mya. It is a modern amphibian separated from the first amphibious tetrapods by the Great Permian–Triassic extinction 252 mya. *S. gardineri* has neither a middle ear nor a collumela ossicle, but its hearing likely allowed for croaking as social vocalization. Recent study has shown that *S. gardineri* had otic vesicles separated from the floor of the mouth by a tissue that is only a few tens of microns thick and the air contained in its oral cavity may have aided in resonation of sound (Boistel et al., 2013). Thus it appears possible that a diverticulum filled with compressible air lying in contact with the otic vesicle could be a sufficient mechanism for transmission of sound waves to be transmitted to the fluids of the inner ear in the absence of any ossicles.

This condition is important as a model for the evolution and development of ossicles from human embryonic pharyngeal arches.

#### 3.3.3 The formation of the external auditory canal conventionally derives from the first pharyngeal slit between the first two embryonic pharyngeal arches

In mice, the external auditory canal appears, however, as a structure derived from the first pharyngeal arch and not from the first pharyngeal slit separating the first two arches (Minoux et al., 2013). The development of the external auditory canal indeed begins with the proliferation of an ectodermal bud from the first arch which comes into contact with the bottom of the first pharyngeal pouch, then opens into a canal whose epithelium of the blind fundus participates in the formation of the membrane separating the external auditory canal and the tympanic cavity (Anthwal & Thompson, 2016). Therefore, if the diverticulum at the origin of the Eustachian tube and the tympanic cavity forms from the first pharyngeal pouch, we can hypothesize that the tympanic membrane is formed, like its primordial formation assumed in *Eusthenopteron*, at the interface between the endodermal layer of the first pharyngeal pouch and the ectodermal bud from the first arch. The hypothesis would then be that the malleus, as well as the fibrous layer of the *pars tensa* but also the tensor tympani innervated by the nerve of the first arch (a branch of the trigeminal nerve) would originate in mammals from the mesoderm of the first arch. These hypotheses could actually be tested. It would follow that the second arch, classically but probably falsely called “mandibular” arch, would be at the origin of the incus, and perhaps even of the very discreet third sheet of mesenchyme of the *pars flaccida* observed in electronic microscopy (Lim, 1968). The majority of authors agree that the second arch serves as the developmental origin of the stapes and its muscle (the stapedius) innervated by the facial nerve (VII).

The evo‐devo concept of the tubotympanic cavity presented in this paper might thus raise some new questions and require further explanations. For example, the phylogeny of the Eustachian tube cartilage is at my knowledge not well known and a more precise description of its development could also make it possible to better understand the origin of the muscles of the Eustachian tube and soft palate, in particular the origin of the internal (levator veli palatini) and external (tensor veli palatini) peristaphyline muscles, which also find their origin in the mesoderm of the pharyngeal arches. The soft palate is indeed a fundamental muscular structure whose components have arisen from pharyngeal arches and underwent immense amounts of modification over evolutionary history in response to the functional demands of tubal ventilation, swallowing, and spoken language (in humans). Yet, despite its vital importance and long evolutionary history, the embryologic development of the soft palate is not yet fully understood.

## 4 CONCLUSION

The nasopharynx is a centrally located space whose boundaries include the posterior nares, the soft palate, basicranium, pharyngeal constrictor muscles, and pharyngotympanic (Eustachian) tubes. From an evo‐devo point of view, its boundaries seem to have arisen from the evolutionary bricolage of gill and airway structures. The bricolage of evolution took millions of years to transform the gill and pharynx of chordates and early vertebrates into the human pharynx, by de novo creating the respiratory nose and transforming the embryonic pharyngeal arches. Indeed, changes in these structures even continued more recently into the evolution of our own species as our supralaryngeal vocal tracts became capable of speech (Bluestone, 2008; Laitman et al., 1979; Pagano & Laitman, 2015). In this way, the evolutionary story of the nasopharynx is the story of our origins.
